# Low‐grade chronic inflammation and immune alterations in childhood and adolescent cancer survivors: A contribution to accelerated aging?

**DOI:** 10.1002/cam4.3788

**Published:** 2021-02-19

**Authors:** Joanna Sulicka‐Grodzicka, Andrzej Surdacki, Michał Seweryn, Tomasz Mikołajczyk, Krzysztof Rewiuk, Tomasz Guzik, Tomasz Grodzicki

**Affiliations:** ^1^ Department of Rheumatology Jagiellonian University Medical College Krakow Poland; ^2^ 2^nd^ Department of Cardiology Jagiellonian University Medical College Krakow Poland; ^3^ Center for Medical Genomics OMICRON Jagiellonian University Medical College Krakow Poland; ^4^ Department of Cancer Biology and Genetics Center for Pharmacogenomics College of Medicine The Ohio State University Columbus OH USA; ^5^ Department of Internal and Agricultural Medicine Jagiellonian University Medical College Krakow Poland; ^6^ Department of Internal Medicine and Gerontology Jagiellonian University Medical College Krakow Poland

**Keywords:** aging, C‐reactive protein, inflammation, lymphocytes, pediatric cancer survivors

## Abstract

**Background:**

The long‐term consequences of chemotherapy and radiotherapy result in a high prevalence and early onset of age‐related chronic diseases in survivors. We aimed to examine whether childhood and adolescent cancer survivors (CS) demonstrate biomarkers of accelerated aging.

**Methods:**

We evaluated 50 young adult CS at 11 [8–15] years after cancer diagnosis, and 30 healthy, age and sex‐matched controls, who were unexposed to cancer therapy. Using a machine‐learning approach, we assessed factors discriminating CS from controls and compared selected biomarkers and lymphocyte subpopulations with data from the Framingham Heart Study (FHS) cohort and the Genotype Tissue Expression (GTEx) project.

**Results:**

Survivors compared with controls had higher levels of C‐reactive protein and fibrinogen. The surface expression of CD38 on T cells was increased, and there was an increase in the percentage of memory T cells in survivors, compared with the unexposed group. The relationships between above cell subpopulations and age were consistent in CS, FHS, and GTEx cohorts, but not in controls.

**Conclusions:**

Young pediatric cancer survivors differ from age‐related controls in terms of activation of the adaptive immune system and chronic, low‐grade inflammation. These changes resemble aging phenotype observed in older population. Further research in biomarkers of aging in young, adult childhood cancer survivors is warranted, as it may facilitate screening and prevention of comorbidities in this population.

## Graphical abstract

Young, adult pediatric cancer survivors are different from age‐matched controls in terms of activation of the adaptive immune system and chronic, low‐grade inflammation. These changes resemble aging phenotype observed in older population.

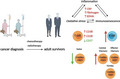

## 1 INTRODUCTION

Pediatric cancer survivors develop chronic conditions prematurely and face a more significant disease burden than age‐matched individuals never diagnosed with cancer. The prevalence of chronic diseases among childhood cancer survivors in their 20 s is similar to that of siblings in their 50 s.^1^ Both cancer itself and treatment‐related long‐term consequences contribute to accelerated aging,^2^ leading to premature aging phenotype in young cancer survivors.^3^, ^4^, ^5^ Aging is associated with a chronic low‐grade inflammation and an accumulation of senescent cells with a pro‐inflammatory senescence‐associated secretory phenotype.^6^ Age‐related changes in the immune system, the reduction in the frequency of naive T cells, increasing prevalence of activated, terminally differentiated, effector memory lymphocytes, and antigen‐experienced memory B cells may contribute to the accumulation of senescent cells.^7^, ^8^ The particular cluster of differentiation (CD) markers of the immune cells participates in the regulation of the immune response. During the activation process, the earliest activation markers are CD69 and CD25.^9^ Expression of CD38 and HLA‐DR is associated with late activation of mature T cells and production of pro‐inflammatory cytokines.^10^

Rate of aging in the general population is controlled by genetic and biochemical processes. The potential hallmarks of aging include genomic instability, telomere attrition, epigenetic alterations, and cellular senescence among the others.^11^ In cancer survivors, exposure to chemotherapy and radiotherapy in childhood and adolescence during the development period may also accelerate aging by affecting one or more of these processes and increase the risk of frailty. St. Jude Lifetime Cohort Study reports frailty in about 8% of survivors at a median age of 33 years,^12^ similar to rate described in adults from the general population aged over 65.^13^

Thus, while the risks of early onset of chronic, age‐related conditions and frailty are increased in childhood cancer survivors, the mechanisms of this relationship remain elusive. Chronic, dysregulated inflammation may represent an essential mechanism of frailty and accelerated aging.^14^ The effect of chronic inflammation on frailty development may surpass the effect of age.^15^ Persistent low‐grade inflammation characterized by an increase in the production of pro‐inflammatory cytokines and inflammatory markers may contribute to the early onset of age‐related diseases.^16^ Cancer chemotherapy may well result in long‐term immune alterations due to epigenetic remodeling of immune cells providing the mechanism of increased development of inflammatory and age‐related diseases in childhood and adolescent cancer survivors. It has been previously shown that pro‐inflammatory cytokines can affect DNA methylation. The initial inflammatory response to genotoxic agents as radiation and alkylating agents may contribute to epigenetic alterations of immune cells and lead to aberrant methylation, which accelerates the process of premature biological aging in young cancer survivors.^17^

The Framingham Heart Study (FHS), since the beginning in 1948, has been dedicated to the recognition of the common factors that contribute to cardiovascular diseases in three generations of participants.^18^ The Genotype‐Tissue Expression (GTEx) project on the other hand is a database and associated tissue bank established to study the relationship between genetic variation and gene expression in human tissues.^19^

We hypothesized that long‐term childhood and adolescent cancer survivors (CS) demonstrate persistent immune alterations of the immune cell subpopulations, resulting in chronic low‐grade inflammation similar to changes observed with aging. We used the group of healthy controls as well as the publicly available data from the FHS and GTEx studies to compare selected biomarkers and lymphocyte subpopulations with the results in young adult cancer survivors.

## 2 MATERIALS AND METHODS

### 2.1 Subjects

We recruited consecutive, young adult childhood and adolescent cancer survivors attending follow‐up clinic at the University Hospital in Kracow, Poland. Of 72 survivors eligible for participation in the study, 50 had completed evaluation. Eligibility criteria included diagnosis of malignancy before 18 years of age; 5 or more years since completion of cancer treatment; and remission. Non‐participants either declined to participate or were not enrolled due to exclusion criteria. Exclusion criteria contained: time from the end of therapy for malignancy shorter than 5 years, relapse or secondary cancer at the time of the study or during 5 preceding years, acute infection, any self‐reported, relevant coexisting diseases (e.g., chronic inflammatory diseases, hypertension, diabetes, chronic kidney disease), or observed significant abnormalities in routine blood or urine assays by discharge letters, and/or other available medical records. Treatment regimens for malignancy were based on the protocols used at different time periods between the years 1995 and 2009 (Table S1). We used medical records to obtain data on demographics, date of diagnosis, date of the end of therapy and treatment protocol. A control group of 30 age‐ and sex‐matched healthy volunteers was enrolled using the same inclusion and exclusion criteria, except for the cancer history. The study was conducted in accordance with the Declaration of Helsinki, and the protocol was approved by the Ethics Committee of Jagiellonian University Medical College (KBET/329/B/2012). Informed consent was obtained from all individual participants included in the study.

### 2.2 Data from the Framingham Heart Study and the GTEx Project

Expression data and phenotypes were accessed from the Framingham Heart Study (FHS) via database of Genotypes and Phenotypes Project (dbGaP). Clinical characteristics of the FHS sample and accession numbers are presented in Table S2. mRNA expression levels have been determined using the Affymetrix Human Exon 1.0 ST array in RNA from whole blood. Clinical phenotypes for the GTEx project were accessed via gtexportal.org. Expression levels of mRNA have been determined using RNA sequencing.

### 2.3 Biochemical assays

Fasting blood samples were collected on the day of clinical evaluation. Serum levels of high‐sensitivity C‐reactive protein (CRP), fibrinogen, complete blood count, plasma lipid levels were measured within 4 hours of venipuncture (Cobas 6000/8000 analyzer, Roche Diagnostics, Indianapolis, IN, USA). For extended biochemical analyses, blood samples were centrifuged at 2000 rpm for 10 min, and plasma was separated and frozen at −70ºC until assayed. Commercially available enzyme‐linked immunosorbent assays were used to measure plasma levels of interleukin (IL)‐6, pentraxin‐3 (R&D Systems, Abingdon, UK), as well as asymmetric dimethylarginine (ADMA) and symmetric dimethylarginine (SDMA) (DLD Diagnostika GmbH, Hamburg, Germany).

### 2.4 Flow cytometry

Blood samples for flow cytometric studies were processed as previously described.^20^, ^21^ Additional details are provided in Table S3. To assess the percentage of T regulatory cells (Treg), CD4+CD25+FoxP3+ cells were gated from CD3+ T lymphocytes. B cells were gated from lymphocytes according to CD19 expression. Then, cells were analyzed for IgD/CD27 expression. This separation allows to distinguish naïve B cells (IgD+CD27‐), memory unswitched B cells (IgD+CD27+), switched memory B cells and plasma cells (both IgD‐CD27+) and double negative B cells (IgD‐CD27‐). CD19+ B cells were also subsequently analyzed for differential expression of CD24/CD38 antigens.

### 2.5 Statistical analysis

Data are presented as medians and interquartile range (25^th^ and 75^th^ percentiles) for continuous parameters, and numbers (proportions) for categorical data. Intergroup differences were assessed by Wilcoxon rank sum test for continuous variables and Fisher's exact test for proportions. To assess the potential relationship between age of cancer diagnosis and inflammatory markers, standard regression analysis was applied with adjustment to the participants’ gender and radiotherapy. Initial statistical tests were performed using STATISTICA, version 9.1. (StatSoft, Inc., Tulsa, Oklahoma, USA).

The “null” linear model of dependency between the given feature, and sex and age was trained on the set of controls and was used to obtain residuals for the given feature in both controls and cancer survivors. These residuals were used in the logistic elastic‐net approach. The most robust features which discriminated between the two study groups were chosen by means of boot strapped five‐fold cross‐validation. The selected residuals were re‐tested for significance using Wilcoxon signed‐rank test. The comparison of the residuals for cancer survivors and controls with the individuals in the FHS cohort was performed by means of an analogos approach, in which we regressed out the effect of sex and age in the FHS cohort participants separately (using a model trained on the FHS cohort) and compared with the residuals obtained above for the in‐house data. For each of the selected markers, the comparison was performed using the Wilcoxon signed‐rank test. Importance index and feature importance score (FIS) analysis were carried out as described previously,^22^ using R‐package “divo” (https://CRAN.R‐project.org/package=divo). A flow chart of the analysis is shown in Figure S1. Wilcoxon test was used to find immune marker combinations with positive FIS.

Deconvolution of immune cell populations using RNA expression was done via xCell software (https://xcell.ucsf.edu). We tested for association between the scores for cell populations and age using standard linear models (with additional covariate: sex). In CS and controls we investigated the association between the percentage of cells and age, and time to end of treatment with standard Pearson product‐moment correlation coefficient, and re‐tested with Spearman rank‐based correlation coefficient. Additionally, the associations between the selected biomarkers (CRP and SMDA) with circulating miRNAs attributed to aging were tested. We chose a list of miRNAs, which were previously attributed to aging and inflammation.^23^, ^24^ From the list of 42 miRNAs, we identified 18 that were expressed in a sufficient number of samples (below 25% of missing values). We used the elastic‐net approach to select the strongest, and the most stable predictors of the CRP and SDMA levels.

## 3 RESULTS

Survivors were studied at the median age of 22 [21–23] years, 50% of the patients were females, 36% were diagnosed in adolescence, the mean time from the diagnosis of cancer was 11 [8–15] years. The most frequent malignancy in the studied group was Hodgkin lymphoma (HL)–48%, followed by acute lymphoblastic leukemia (ALL)–32%, and non‐Hodgkin lymphoma–12%. Solid tumors (neuroblastoma) accounted for less than 1% of cancers. Overall, 54% of patients underwent radiotherapy, including 5 patients with central nervous system radiotherapy for ALL. In HL patients, involved field radiotherapy was used in 83% of patients. CRP and fibrinogen were significantly higher in survivors compared with healthy controls. None of the patients were treated with bone marrow transplantation. In regression analysis, age at diagnosis was positively associated with CRP (beta=0.38 ± 0.14; *p* = 0.008), independently of sex and radiotherapy. The regression model was statistically significant (R^2^=0.21; *p* = 0.01). Clinical and biochemical characteristics of study participants are presented in Table 1.

**TABLE 1:** Clinical characteristics of survivors and controls

	CS (*n* = 50)	Controls (*n* = 30)	*p*‐value
Age, years	22 [21–23]	23 [22–26]	0.032
Age at diagnosis, years	13 [8–15]	N/A	N/A
Time to end of treatment, years	9.8 [7.5–13.8]	N/A	N/A
Sex (female), n (%)	25 (50)	14 (46.7)	0.775
Smoking, n (%)	8 (16)	3 (10)	0.451
WHR	0.82 [0.76–0.88]	0.79 [0.74–0.84]	0.121
BMI, kg/m^2^	22.3 [20.6–27.4]	22.3 [21.0–23.9]	0.450
LDL, mmol/L	2.5 [2.0–3.1]	2.0 [1.9–2.6]	0.049
HDL, mmol/L	1.7 [1.3–1.9]	1.6 [1.4–1.9]	0.709
Total cholesterol, mmol/L	4.6 [4.1–5.0]	4.2 [3.8–4.6]	0.025
Leukocytes, 10^3^/µl	5.47 [4.73–6.51]	5.54 [4.71–6.74]	0.874
Neutrophils, 10^3^/µl	3.2 [2.6–3.9]	2.9 [2.4–3.7]	0.110
Lymphocytes, 10^3^/µl	1.6 [1.4–1.9]	1.9 [1.5–2.3]	0.012
Monocytes, 10^3^/µl	0.5 [0.4–0.6]	0.6 [0.4–0.7]	0.078
Platelets, 10^3^/µl	217 [181–243]	236 [199–260]	0.065
hsCRP, mg/L	0.79 [0.33–2.42]	0.31 [0.17–0.57]	0.007
Fibrinogen, g/L	2.8 [2.5–3.3]	2.2 [1.9–2.5]	<0.001
Interleukin−6, pg/ml	0.69 [0.49–0.99]	0.71 [0.50–0.86]	0.963
Pentraxin 3, ng/ml	1.1 [0.6–5.9]	1.2 [0.8–1.7]	0.745
SDMA, ng/ml	118 [99–134]	104 [89–118]	0.014
ADMA, µmol/L	0.36 [0.33–0.43]	0.36 [0.33–0.42]	0.963

Data are presented as medians [interquartile range] and percentages for categorical variables.

Abbreviations: ADMA, asymmetric dimethylarginine; BMI, body mass index; CS, cancer survivors; HDL, high density cholesterol; hsCRP, high sensitivity C‐reactive protein; LDL, low density cholesterol; N/A, not applicable; SDMA, symmetric dimethylarginine; WHR, waist to hip ratio.

**FIGURE 1:**
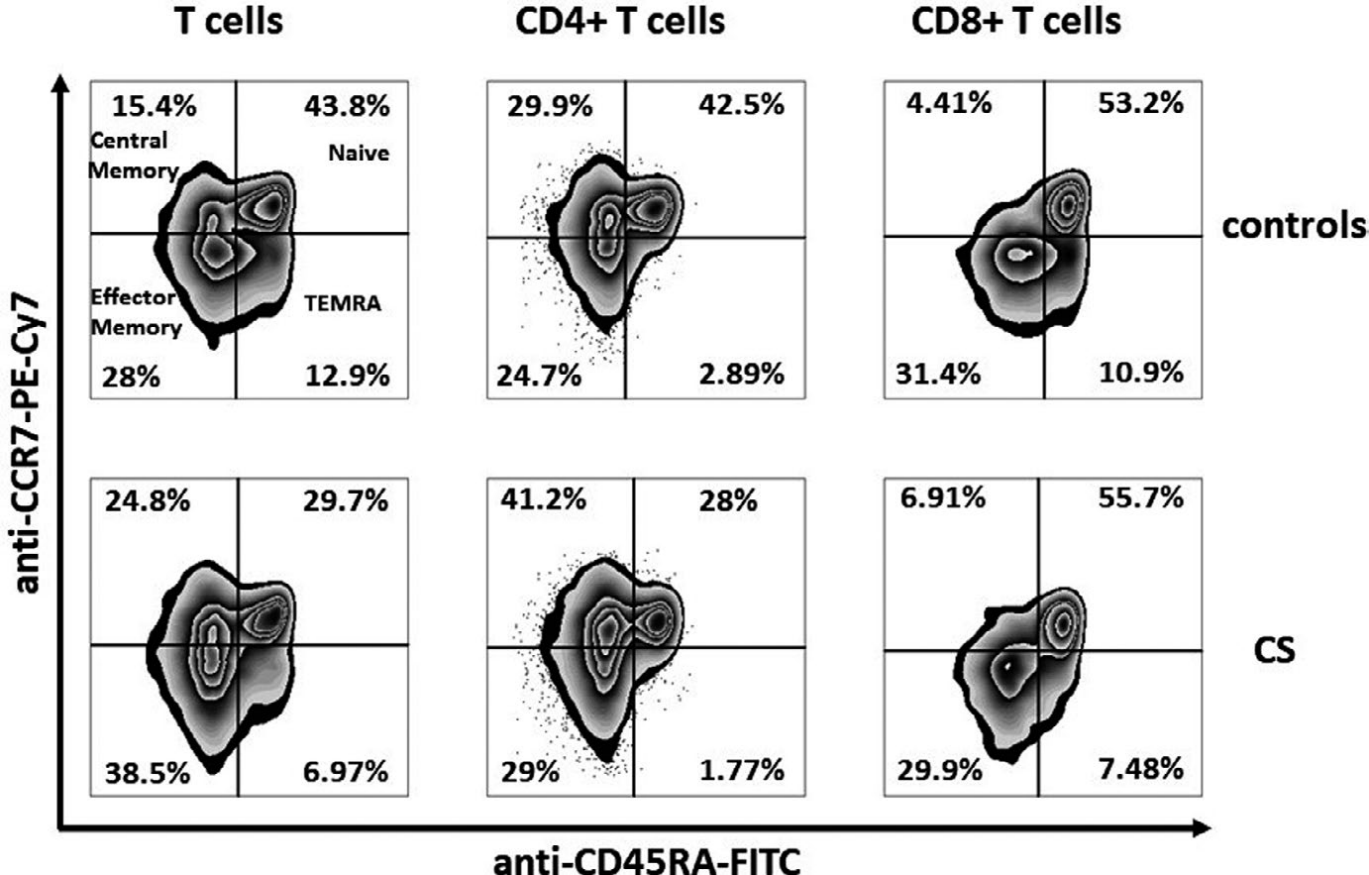
Comparison of T lymphocytes and T‐cell subpopulations in cancer survivors and controls. Representative plots with cells obtained from a childhood cancer survivor patient, and a patient from the control group showing decreased percentage of naive T cells and increased percentages of central memory and effector memory T cells in cancer survivor (CS). (A) T lymphocytes; (B) CD4+ T lymphocytes; (C) CD8+ T lymphocytes

There was a reduction in the percentage of naive T lymphocytes and increase in the percentage of effector memory T cells, and central memory T cells in survivors, compared with the unexposed group. The analysis of CD4+ and CD8+ subsets revealed that above alterations were observed particularly for CD4+ lymphocytes (Table 2, Figure 1). Among CD4+ T cells, the percentages of CD25+FoxP3+ (regulatory T cells) were comparable between the groups (7.1 [5.9–8.0] vs. 7.2 [6.2–8.3] %; *p* = 0.46) in survivors and controls, respectively.

**TABLE 2:** Comparison of T cells subsets (CD4+, CD8+) and subpopulations (naive, central memory, effector memory and TEMRA) within each subset in cancer survivors and controls

		T cells	CD4+ T cells		CD8+ T cells
naive (%)	CS	37.5 [29.7–45.4]	38.3 [30.7–49]	43.1 [29.3–54.3]	
	Controls	43.6 [41.2–48.9]^b^	50 [41.6–55.5]^b^		47.7 [39.9–56.7]
central memory(%)	CS	19.7 [15.7–21.7]	31.3 [24.8–35.8]		5.0 [3.7–6.9]
	Controls	15.4 [12.7–18.4]^b^	24 [21.4–27.6]^c^		4.2 [3–4.6]^a^
effector memory (%)	CS	29.7 [26–37.6]	25.6 [20.8–31.9]		38.4 [29.6–44.1]
	Controls	26.8 [22.3–30.4]^a^	20.8 [17.1–27.4]		34.8 [27.2–38.5]
TEMRA(%)	CS	9.4 [7.8–14.4]	2.7 [2.4–3.4]		10.6 [7–21]
	Controls	10.4 [7.6–16]	3.4 [2.9–4.2]^b^		10.9 [8.3–18.5]

Data are presented as medians of percentages [interquartile range] of T‐cell subpopulations.

Abbreviations: CS, cancer survivors; TEMRA, terminally differentiated effector memory.

^a^ *p*<0.05.

^b^ *p*<0.01.

^c^ *p*<0.001.

The percentages of lymphocytes T and subpopulations expressing activation markers were comparable except for CD38 (34 [28.6–42.8] % in survivors vs. 36.6 [34.3–46.9] % in controls; *p* = 0.044). The median surface expression of activation marker CD38 on T cells in CS was 1.2‐fold larger compared with controls (707 [601–847] vs. 579 [532–634] (expressed as mean fluorescence intensity [MFI]); *p* < 0.001). The analysis of individual T cell subsets has shown that the expression of CD38 was increased predominantly on CD8+ lymphocytes (1.7‐fold increase), and it remained similar in both groups on CD4+ lymphocytes. There was no significant differences between the survivors and controls in the expression of early activation marker CD69 on T cells. The expression of CD57 on CD8+ lymphocytes in the studied group was lower than in the healthy controls (0.7‐fold decrease; 24227 [16627–33783] vs. 33687 [24152–41499] MFI; *p* < 0.01), while the expression of CD28 on both subsets of T cells was increased in comparison with the control group (1.2‐fold increased expression on CD4+ lymphocytes, 1.1‐fold increased expression on CD8+ lymphocytes). (Table S4, Figure S2).

There was an increase in the percentage of total CD19+ B lymphocytes, and naive B cells in survivors in comparison with controls, while the percentage of double negative B cells was decreased in CS when related to the control group. The population of CD24‐CD38‐ B cells, both naïve and memory, was significantly decreased in survivors (Table 3).

**TABLE 3:** Distribution of B‐cell subsets in cancer survivors and controls

		CS	Controls	*p*‐value
B cells (%)	CD19	11.5 [9.3–14.6]	6.7 [5.5–7.5]	<0.001
Naive (%)	CD27‐IgD+	74.8 [62.6–81.9]	67.8 [60.9–75.3]	0.030
	CD27‐IgD+CD24‐CD38‐	0.8 [0.5–1.3]	1.7 [1–2.7]	<0.001
Transitional (%)	CD27‐IgD‐CD24+CD38+	30.4 [21.0–39.1]	24.6 [19.3–28.3]	0.121
Memory unswitched (%)	CD27+IgD+	8.6 [6.3–11.8]	10 [6.6–13.1]	0.44
	CD27+IgD+CD24‐CD38‐	1.3 [0.5–2.3]	0.7 [0.3–1.8]	0.369
Memory switched (%)	CD27+IgD‐	11.2 [8.8–17.2]	14.6 [10.1–18.9]	0.068
	CD27+IgD‐CD24‐CD38‐	4.2 [2.3–5.4]	5.3 [4.5–7.1]	0.003
Double negative (%)	CD27‐IgD‐	4.1 [2.8–5.5]	6.9 [4.2–8.7]	<0.001
Plasma cells (%)	CD27+IgD‐CD24‐CD38+	5.8 [3.2–8.5]	3.9 [2.3–5.5]]	0.087

Data are presented as medians of percentages of cell populations [interquartile range].

Abbreviation: CS, cancer survivors.

**FIGURE 2:**
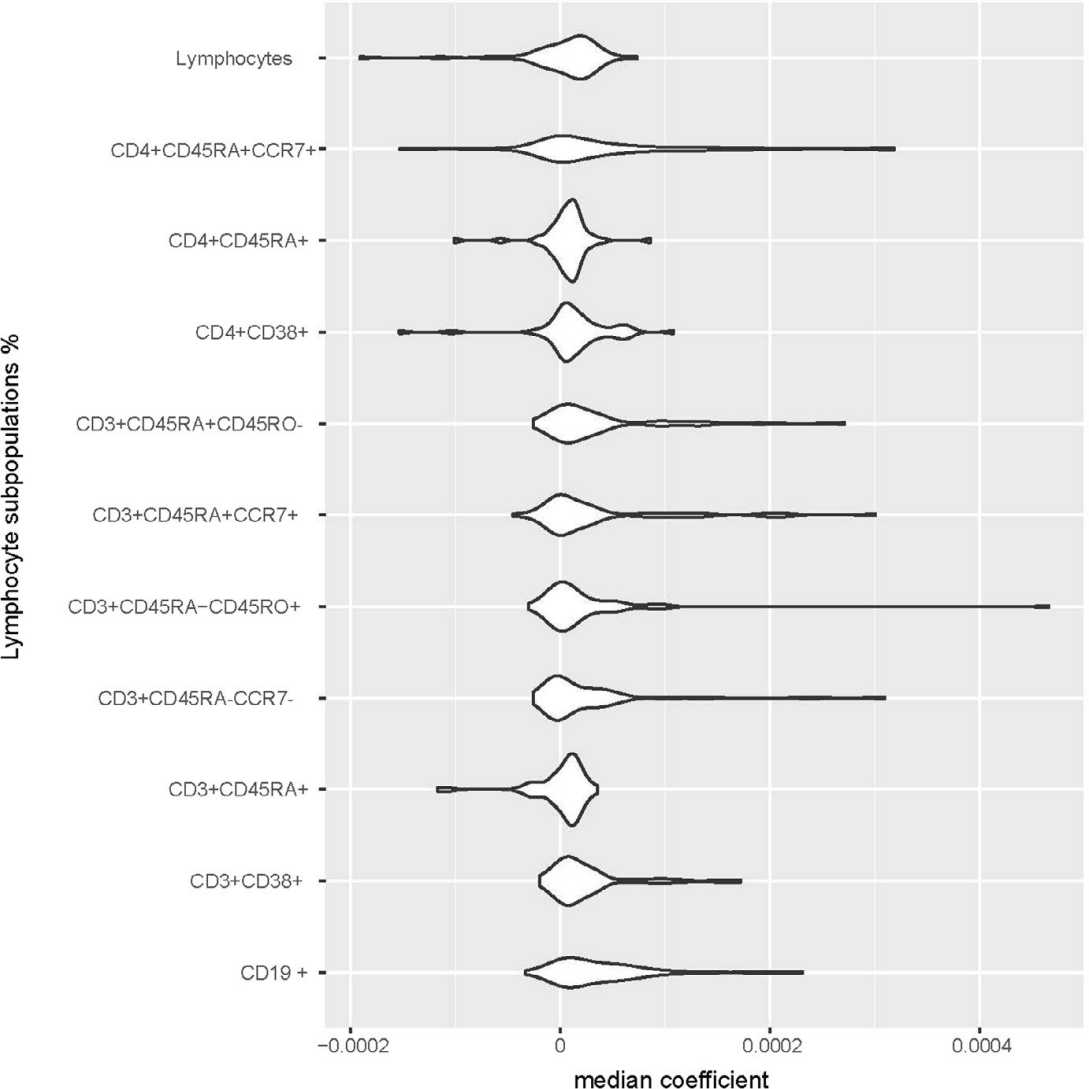
Violin plot showing contribution of selected lymphocyte subpopulations discriminating cancer survivors from controls. Coefficients were obtained from the machine‐learning approach based on FIS (see Table S2)

To investigate the effect of sex and age on the biochemical parameters in the two groups, we trained a linear model on the control group to regress‐out the effect of age and sex, and subsequently used this model to obtain residuals for the variable of interest in the CS group. Subsequently, we used a machine‐learning approach to detect the variables which best discriminated between controls and CS. We applied an elastic‐net regression model to identify parameters associated with cancer survivorship. Table 4 lists variables selected by elastic net regression model, and median coefficient for each parameter. Furthermore, using machine‐learning approach based on FIS we have shown that naive and memory T lymphocytes, the subpopulation of naive CD4 T lymphocytes, CD4 cells expressing CD38, and CD19 B cells were strongly, positively associated with survivorship. Figure 2 illustrates distribution and probability density of coefficients for selected lymphocyte subpopulations.

**TABLE 4:** Parameters associated with cancer survivorship. Coefficients of parameters extracted by elastic net regression model, and comparison of selected parameters in cancer survivors and Controls with the FHS cohort

	median coefficient (95% confidence interval)	*p*‐value FHS vs. controls	*p*‐value FHS vs. cancer survivors
WHR	15.557 (5.410; 58.991)	N/A	N/A
Neutrophils, 10^3^/µl	0.0003 (0; 0.002)	0.564	<0.001
Lymphocytes, 10^3^/µl	−0.0002 (−0.003; 0)	0.564	<0.001
Monocytes, 10^3^/µl	−0.0001(−0.005; 0)	0.564	<0.001
Platelets, 10^3^/µl	−0.009 (−0.020; −0.0004)	0.961	0.012
MCH, pg	0.396 (0.108; 0.982)	0.964	0.004
MCHC, g/dl	0.336 (0; 0.393)	0.788	<0.001
Fibrinogen, g/L	1.617 (0.875; 8.903)	N/A	N/A
Interleukin−6, pg/ml	−0.518 (−5.292; 0)	0.133	0.352
SDMA, ng/ml	0.017 (0.005; 0.095)	0.061	0.025
Pentraxin−3, ng/ml	0.261 (0; 1.349)	N/A	N/A

Higher absolute value of the coefficient reflects higher contribution to the model.

Abbreviations: FHS, Framingham Heart Study; MCH, mean corpuscular hemoglobin; MCHC, mean corpuscular hemoglobin concentration; N/A, not available or not applicable; SDMA, symmetric dimethylarginine; WHR, waist‐to‐hip ratio.

Analysis of associations between B cells, T cell subpopulations, and age revealed different correlations among CS and controls. We were unable to perform the direct comparison between study groups at the target statistical significance level of 0.05 due to limited sample size. We further re‐tested a few of these populations in the FHS and GTEx project cohorts, where lymphocyte subpopulations were estimated from mRNA expression in whole blood (Table S5). In each of these cases, the direction of correlation was consistent with the direction of association between the current age in CS and the variable of interest, but not with the direction of correlation with age in the control group (Table 5). Time to end of treatment was significantly associated only with the percentage of B cells.

**TABLE 5:** Association between percentages of lymphocyte subpopulations, age, and time to end of treatment

	Correlation with age		Correlation with time to end of treatment
	CS	Controls	CS
Lymphocytes	0.24; *p* = 0.09	0.18; *p* = 0.36	0.44; *p* = 0.23
CD3+CD45RA+	−0.14; *p* = 0.34	0.23; *p* = 0.24	−0.04; *p* = 0.91
CD3+CD45RA‐	0.17; *p* = 0.23	−0.37; *p* = 0.05	−0.01; *p* = 0.97
CD3+CD45RA+CCR7+	−0.27; *p* = 0.06	0.12; *p* = 0.57	−0.18; *p* = 0.64
CD3+CD45RA‐CCR7‐	0.27; *p* = 0.06	−0.27; *p* = 0.16	0.11; *p* = 0.77
CD4+CD45RA+CCR7+	−0.10; *p* = 0.47	0.10; *p* = 0.61	−0.11; *p* = 0.77
CD3+CD38+	−0.09; *p* = 0.53	−0.11; *p* = 0.56	−0.03; *p* = 0.94
CD3+CD45RA+	−0.13; *p* = 0.37	0.28; *p* = 0.13	0.16, *p* = 0.67
CD4+CD38+	−0.15; *p* = 0.30	0.05; *p* = 0.77	0.01; *p* = 0.98
CD4+CD45RA+	−0.08; *p* = 0.58	0.27; *p* = 0.13	0.10; *p* = 0.80
CD19+	−0.09; *p* = 0.53	0.29; *p* = 0.12	−0.83; *p* = 0.02

Data are shown as Pearson's correlation coefficients and *p*‐values.

Abbreviation: CS, cancer survivors.

CRP was associated with two miRNAs: hsa‐miR‐128‐3p (median coefficient: 0.019) and hsa‐miR‐151a‐5p (median coefficient 0.1054), and SDMA was associated with hsa‐miR‐151a‐5p (median coefficient: 0.003) in the 20‐fold cross‐validation analysis.

## 4 DISCUSSION

**FIGURE 3:**
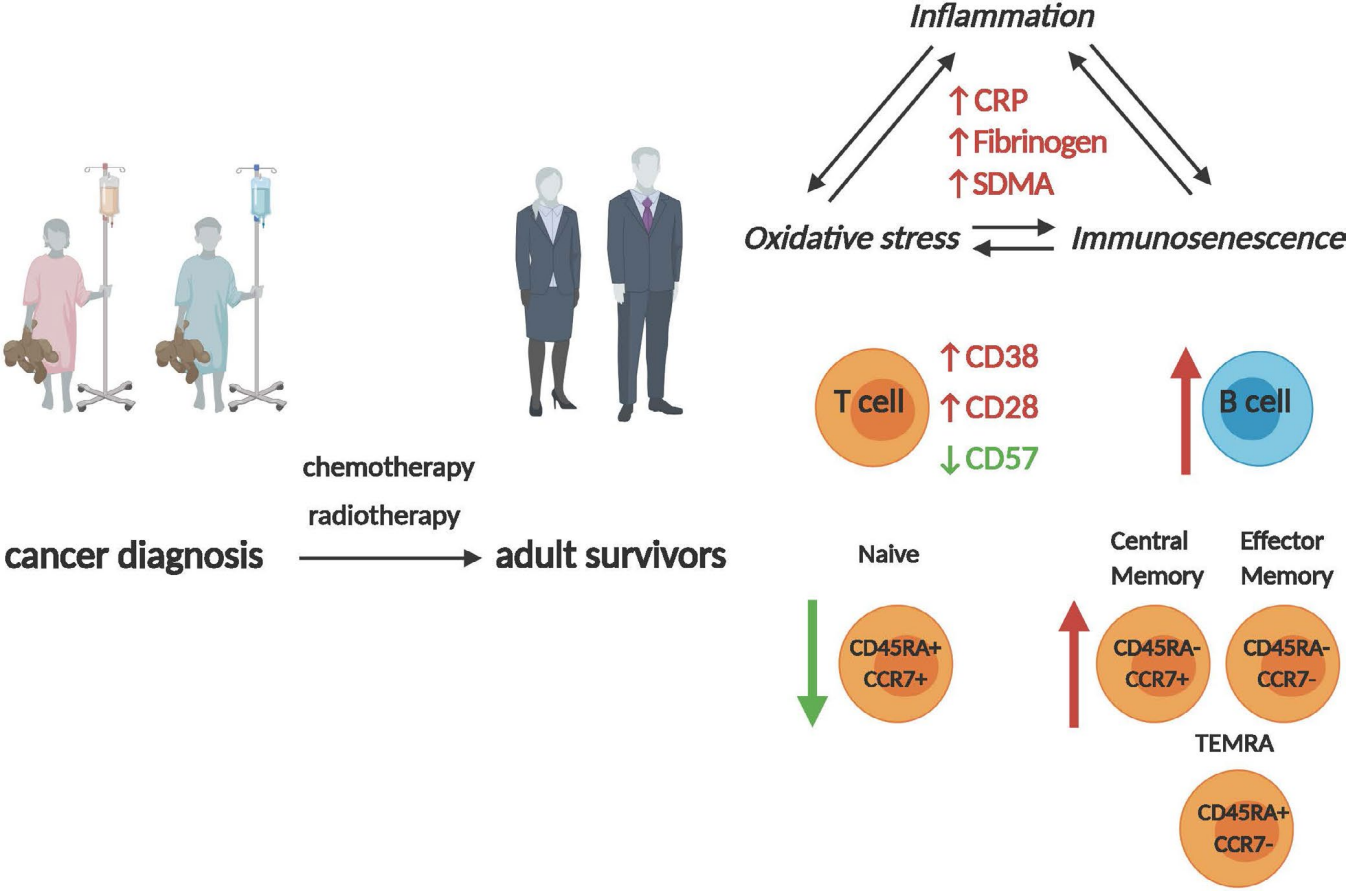
Model of cancer‐related inflammation, oxidative stress, and immunosenescence in cancer survivors. Please refer to the text for the detailed description. The figure was created with BioRender.com

We report that childhood cancer survivors demonstrate a pro‐inflammatory status characterized by increase in inflammatory markers: C‐reactive protein and fibrinogen, as well as a shift toward memory and activated T lymphocytes – a decrease in naive T cells and an increase in memory T cells, and a higher expression of activation marker CD38. Furthermore, we demonstrate dysregulation of B cell lineage in the study group (Figure 3). These changes suggest a chronic immune activation in cancer survivors patients compared with age‐matched individuals. Increasing age at cancer diagnosis was associated with higher CRP in survivors. Interleukin‐6 and CRP levels increase with age and are commonly used as indicators of inflammation,^25^ and chronic low‐grade inflammation in older individuals contributes to the onset of chronic diseases. It has been suggested that this mechanism may be similar, but triggered prematurely in younger childhood cancer survivors who were exposed to cancer treatment early in life.^26^ Cancer treatment can influence many mechanisms of aging, including changes in immune function.^27^ Long‐term immune modulations like changes in the distribution of T cell subsets or in the pathways controlling T cell differentiation or cytokine production may persist following treatment.^17^ In our study the expression of activation marker CD38 on CD8+ T cells was significantly higher in survivors compared with controls. This increase observed in MFI reflects individual cell activation status but was not associated with increased proportion of total CD38+ cells. The main function of CD38 is the regulation of activation and proliferation of human T lymphocytes. A report from Bahri et al. showed that CD8+CD38++ T lymphocytes in humans inhibit CD4+ effector T cell proliferation, and may regulate immune homeostasis during inflammation.^28^ T cells expressing high levels of CD38 have an enhanced cytokine production capability.^10^ A recent report by Azanan et al. was the first to draw attention to a similar immune phenotype in elderly subjects and young adult childhood leukemia survivors.^29^ Similar to this study, they reported elevated CRP and increased percentage of CD8+CD38+ T cells. Additionally, they observed a propensity to increased proportion of CD16+ monocytes. Therefore, these results presumably reflect chronic activation of the innate immunity in childhood leukemia survivors in addition to changes in the adaptive immune compartment which correspond to higher percentages of central memory/effector memory T cells.

Moreover, we found that cancer survivors in our study had significantly higher expression of CD28 and lower expression of CD57 on T cells than age matched controls. CD28 is a co‐stimulatory molecule, which has multiple functions during T‐cell activation, proliferation, and survival.^30^, ^31^ CD28 ligation is essential in promoting proliferation and effector function of conventional T cells, although it also promotes the function of regulatory T cells, including the production of interleukin‐10 (Il‐10).^32^ It is noteworthy that Il‐10 was significantly elevated in young childhood ALL survivors compared with healthy controls.^4^ However, since plasma Il‐10 level was not measured in our study, and the percentages of regulatory T cells (CD4+CD25+FoxP3) were comparable between survivors and controls, the theoretical associations mentioned above would have to be confirmed in further studies. It could be hypothesized though that increased CD28 expression in CS might be a compensatory mechanism, as CD28 expression in human T cells was shown to decrease replicative senescence through reduced secretion of inflammatory cytokines.^33^

In our study, there was a significant increase in total and naive B lymphocytes and decrease of a B cell population defined as CD27‐IgD‐ double negative (DN) B cells in cancer survivors, which is inconsistent with the propensity of naive/memory B‐cell subsets observed in elderly.^34^, ^35^ The reduction of DN B cells in cancer survivors might be a manifestation of dysregulation of the immune system related to cancer and/or cancer treatment. We also found that CD38‐CD24‐ B cells which are predominantly memory B lymphocytes ^36^ were decreased in cancer survivors. Our results might suggest that B cells may not take the key part in the generation or the maintenance of inflammation in cancer survivors. Although inflammation contributes to the development of B cells, the precise mechanisms of changes in B lymphocytes compartment associated with cancer survivorship remain to be elucidated. It has been suggested that chronic inflammation is related to development of age‐related diseases.^37^ Cancer therapy could contribute to long‐term epigenetic changes in immune cells, resulting in increased prevalence of inflammatory diseases in cancer survivors. We have previously reported a pro‐inflammatory state among adult survivors of childhood ALL.^38^, ^39^ An altered DNA methylation signature in T cells at genes involved in inflammatory processes was found in childhood cancer survivors over 10 years following treatment. Moreover, it was associated with increased frequency of T cell‐producing type 1 cytokines and activation of related signaling pathways.^17^

To gain further insight into molecular pathways which may contribute to observed results, we used publicly available data from the FHS and GTEx studies. We found that in FHS two miRNAs related to immunity, inflammation, and aging were strongly associated with levels of CRP and SDMA: miRNA‐128, which is linked to hematopoietic stem cell differentiation ^40^; and miRNA‐151a, which was shown to regulate the expression of STAT3 ^41^ and is highly expressed in B‐cells.^42^ Furthermore, the direction of correlations between the age and the selected lymphocyte subpopulations in FHS and GTEx cohorts corresponded to the direction of associations in CS, but not in the control group. This may indicate that these associations may be much stronger in older individuals, although confounding effects due to differences in study design between the groups must be considered. Data from FHS and GTEx did not constitute a direct reference in our results, rather the associations between relevant clinical and molecular characteristics were used to elucidate the results obtained for the in‐house data. Therefore these initial observations have yet to be confirmed in further studies.

A number of limitations of the study should be acknowledged. First of all, the patients were recruited only from the group attending a single follow‐up clinic. Additionally, patients were treated according to various protocols used between the years 1995 and 2009, and it may be debated if evaluation of late effects of different therapeutic approaches is relevant. However, the central components of chemotherapy treatment have not changed substantially since the 1990 s, and therapy modifications included mainly refinements of protocols using agents that have been available for many years.^43^ Another limitation is the fact that the results of analyses of large datasets may be affected by biological sources of variation such as age, sex, ethnicity, or batch effects. We were not able to exclude all sources of variation, we regressed out the variation due to effects of age and sex in the FHS cohort, although there were still some significant confounding effects due to differences in study design between the in‐house group and the publicly available cohorts. The next limitation of the study is the lack of functional assays, which would complement the flow cytometric data describing the phenotype of the immune cells and could provide the additional insight into the cell functionality. Recently published data revealed that in cancer, T cells become dysfunctional due to the persistent antigen exposure. Dysfunctional T cells have reduced proliferative capacity, decreased effector function, and overexpression of multiple inhibitory receptors.^44^ As mentioned above, it cannot be excluded that chemotherapy and radiotherapy may influence T‐cell phenotype even years after completing the therapy.^45^ Low‐grade inflammation and altered immune function have been found in survivors treated with HSCT (hematopoietic stem cell transplantation). These survivors in full remission had an altered DNA methylation signature in T cells, particularly at genes controlling oxidative stress and inflammatory processes. Functional assay on T cells stimulated with PMA/ionomycin revealed Th1 phenotype and higher production of interferon‐γ and tumor necrosis factor‐α in cancer survivors treated with HSCT in comparison with non‐irradiated subjects.^17^ Finally, we were not able to control for all the additional factors that could affect the results. Nevertheless, in young survivors of a childhood‐onset cancer, environmental exposures and lifestyle factors may have a lesser effect on the process of biological aging compared with adult survivors with chronic diseases and lifelong accumulation of various stressors. Lifestyle behaviors, such as smoking and alcohol consumption, and risk factors such as obesity were comparable in survivors and controls at the time of the study, except for the higher total cholesterol in CS. However, we did not have data on physical activity, diet, exposure to chemicals, or various infections in the study participants.

In summary, we have shown that healthy, young childhood and adolescent cancer survivors are different from age‐matched controls in terms of activation of the adaptive immune system and chronic, low‐grade inflammation, which is relatively similar to aging phenotype observed in older population. Our findings suggest that the immunosenescent phenotype (i.e., decreased pool of naïve lymphocytes and accumulation of memory/effector cells with reduced proliferative ability) in childhood cancer survivors is mainly attributed to T cells, but dysregulation of B‐cell lineage is also present. Whether those observations may apply to general population of pediatric cancer survivors remains to be confirmed in larger studies.

## CONFLICTS OF INTEREST

The authors declare no conflict of interest.

## Supporting information

Fig S1

Fig S2

Table S1

Table S2

Table S3

Table S4

Table S5

## Data Availability

The data that support the findings of this study are available from the corresponding author upon reasonable request. The expression and phenotypes data were accessed from the Framingham Heart Study (FHS) via database of Genotypes and Phenotypes Project (dbGaP) #5358 accession number phs000007. Data were accessed from the following datasets: pht002234, pht002889, pht00476, pht003794. These data were derived from the following resource available in the public domain: http://www.ncbi.nlm.nih.gov/projects/gap/cgi‐bin/study.cgi?id=phs000007. Clinical phenotypes for the GTEx project were accessed via gtexportal.org.
