# Supplementary figures and images for: Low‐grade chronic inflammation and immune alterations in childhood and adolescent cancer survivors: A contribution to accelerated aging?
Source: Cancer Med. 2021 Feb 19;10(5):1772–82. doi: 10.1002/cam4.3788 (PMC7940211; doi:10.1002/cam4.3788)

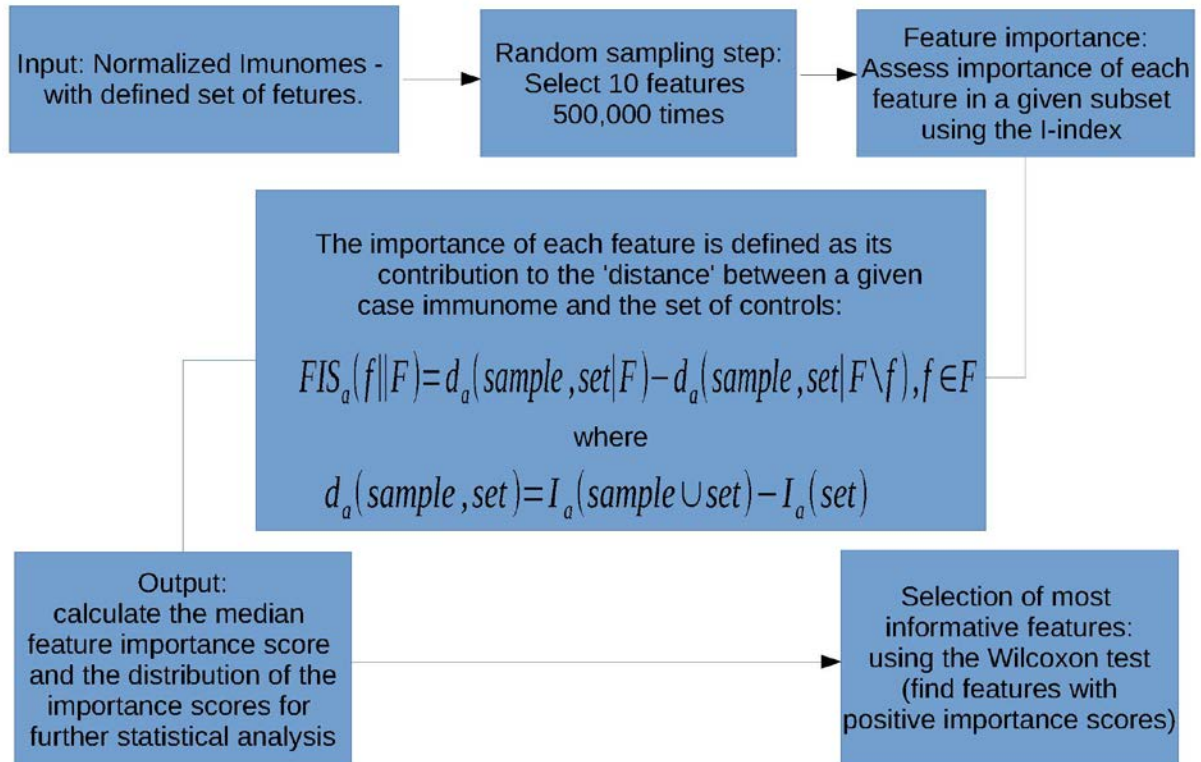

Figure S1. Feature selection algorithm. FIS, feature importance score.

Supplement: Supplementary file 1 — Fig S1 [file CAM4-10-1772-s002.pdf]
