# Supplementary material for: Low‐grade chronic inflammation and immune alterations in childhood and adolescent cancer survivors: A contribution to accelerated aging?
Source: Cancer Med. 2021 Feb 19;10(5):1772–82. doi: 10.1002/cam4.3788 (PMC7940211; doi:10.1002/cam4.3788)
Supplement: Supplementary file 2 — Fig S2 [file CAM4-10-1772-s003.pdf]

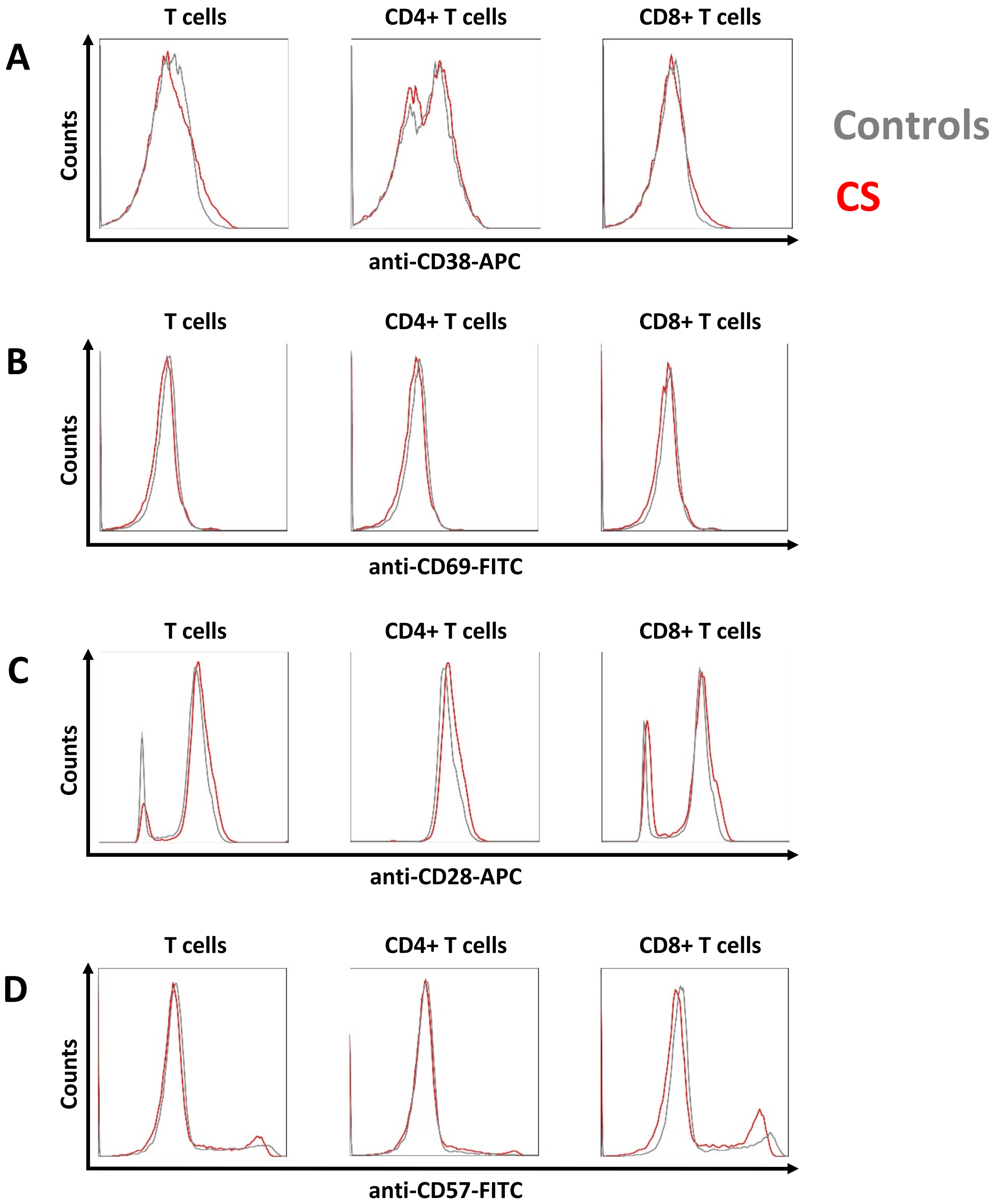

A
B
C
D
T cells
CD4+ T cells
CD8+ T cells
Counts
anti-CD38-APC
anti-CD69-FITC
anti-CD28-APC
anti-CD57-FITC
Controls
CS

Figure S2. Flow cytometric examples of the expression of CD38 (Panel A), CD69 (Panel B), CD28 (Panel C) and CD57 (Panel D) on T cells and their subsets CD4+ and CD8+. CS, cancer survivors.
