# Supplementary material for: Low‐grade chronic inflammation and immune alterations in childhood and adolescent cancer survivors: A contribution to accelerated aging?
Source: Cancer Med. 2021 Feb 19;10(5):1772–82. doi: 10.1002/cam4.3788 (PMC7940211; doi:10.1002/cam4.3788)
Supplement: Supplementary file 3 — Table S1 [file CAM4-10-1772-s001.docx]

**Table S1.** Treatment protocols used in the treating institution between the years 1995 and 2009.

| Malignancy | Protocol | Time period,  years | Chemotherapy | Radiotherapy |
| --- | --- | --- | --- | --- |
| Hodgkin lymphoma | HD-94 | 1994-1997 | MVPP:  nitrogen mustard, vinblastine, prednisone, doxorubicin;  B-DOPA: bleomycin, decarbazine, vincristine, prednisone, doxorubicin | involved- fields (20-30 Grey); except low risk group in HD-97 (7%) |
|  | HD-97 | 1998-2009 |  |  |
| Non-Hodgkin lymphoma | B-NHL-BFM90 (with modifications of PPLLSG)  LCAL 93  EURO-LB 02 | since 1995 | dexamethasone, methotrexate, cytarabine, prednisolone, vincristine, ifosfamide, cyclophosphamide, etoposide, doxorubicine,  daunorubicin,  L-asparaginase,  6-mercaptopurine | cranial radiotherapy (12-18 Grey) in stage III/IV; no involved-field radiation/ considered in non-responders |
| Acute lymphoblastic leukemia | PGP-ALL-95  New York  ALLIC- 2000 | 1995-2003  1987-2003  since 2003 | prednisone, vincristine,  L-asparaginase, daunorubicin, methotrexat,  6-mercaptopurine, cytarabine, cyclophosphamide, dexametasone, doxorubicine,  6-thioguanine | cranial radiotherapy in leukemic involvement of CNS/risk factors for CNS relapse;  after 1995: reduced dose/no CNS radiotherapy |

PPLLSG, Polish Pediatric Leukemia/Lymphoma Study Group; PGP, Polish Pediatric Group; LCAL, large cell anaplastic lymphoma; CNS, central nervous system.
