# Supplementary material for: Low‐grade chronic inflammation and immune alterations in childhood and adolescent cancer survivors: A contribution to accelerated aging?
Source: Cancer Med. 2021 Feb 19;10(5):1772–82. doi: 10.1002/cam4.3788 (PMC7940211; doi:10.1002/cam4.3788)
Supplement: Supplementary file 4 — Table S2 [file CAM4-10-1772-s006.docx]

**Table S2.** Selected clinical characteristics of the Framingham Heart Study sample.

|  | **median (range)** | **age, years**  **median (range)** | **sex, n** | |
| --- | --- | --- | --- | --- |
|  |  |  | **male** | **female** |
| Platelets, 10^3^/µl | 247 (130-414) | 29 (24-31) | 100 | 112 |
| MCHC, g/dL | 34 (31.9-35.5) | 29 (24-31) | 100 | 112 |
| MCH, pg | 30.8 924.3-33.9) | 29 (24-31) | 100 | 112 |
| Neutrophils, 10^3^/µl | 3.4 (1.2-7.4) | 29 (24-31) | 100 | 112 |
| Lymphocytes, 10^3^/µl | 1.7 (0.9-3.6) | 29 (24-31) | 100 | 112 |
| Monocytes, 10^3^/µl | 0.5 (0.2-1.0) | 29 (24-31) | 100 | 112 |
| Glucose, mmol/L | 4.9 (4.3-9.9) | 29 (24-31) | 100 | 112 |
| SDMA, µmol/L | 0.93 (0.6-1.52) | 39 (35-41) | 23 | 21 |
| IL-6, pg/ml | 1.2 (0.5-6.0) | 33 (20-38) | 15 | 22 |

MCHC, mean corpuscular hemoglobin concentration; MCH, mean corpuscular hemoglobin, SDMA, symmetric dimethylarginine; IL-6, interleukin 6.

Database of Genotypes and Phenotypes Project (dbGaP) #5358 (dbGaP accession number phs000007). Data were accessed from the following datasets: pht002234, pht002889, pht00476, pht003794. SDMA was analyzed for a subset of individuals aged ≤42 years (n=44, median age 39 (35-41) years, 48% women) from pht002234 (Offspring Cohort, Exam 5). From pht002889 (Generation 3 Exam 2 and New Offspring Spouse Exam 2) the following variables were analyzed for subjects ≤31 years of age: MCH, MCHC, platelets, neutrophils, lymphocytes, monocytes and eosinophils (n=212, median age 29 (24-31) years, women 53%). From pht006026 (Generation 3, New Offspring Spouse, and Omni 2, Exam 1 - Exam 2) fasting blood glucose and HDL were analyzed for individuals aged ≤31 years (n=212, median age 29 (24-31) years, women 53%). From pht002476 (GO-ESP: Heart Cohorts Component of the Exome Sequencing Project) IL-6 was analyzed in individuals aged ≤38 years (n=37, median age 33 (20-38) years, women 59%). For CRP analysis, we applied data for 87 participants (median age 24 (17-26) years, women 59%).

Further information on these phenotypes can be found at http://www.ncbi.nlm.nih.gov/projects/gap/cgi-bin/study.cgi?id=phs000007. Our access of the FHS datasets was approved by the Ohio State University IRB (Protocol #2013H0096).
