# Supplementary material for: Low‐grade chronic inflammation and immune alterations in childhood and adolescent cancer survivors: A contribution to accelerated aging?
Source: Cancer Med. 2021 Feb 19;10(5):1772–82. doi: 10.1002/cam4.3788 (PMC7940211; doi:10.1002/cam4.3788)
Supplement: Supplementary file 5 — Table S3 [file CAM4-10-1772-s004.docx]

**Table S3.** Monoclonal antibodies used in the study

| **Antibody conjugated with fluorochrome** | **Clone** | **Company** |
| --- | --- | --- |
| CD3–PerCP | SK7 | BD Biosciences |
| CD4–APC | SK3 | BD Biosciences |
| CD4–PE-Cy7 | SK3 | BD Biosciences |
| CD8–APC-H7 | SK1 | BD Biosciences |
| CD19–APC-H7 | SJ25C1 | BD Biosciences |
| CD28–APC | CD28.2 | BD Biosciences |
| CD38–APC | HIT2 | BD Biosciences |
| CD45RO–PE | UCHL1 | BD Biosciences |
| CD45RA–FITC | L48 | BD Biosciences |
| CD57–FITC | NK-1 | BD Biosciences |
| CD69–FITC | FN50 | BD Biosciences |
| CCR7(CD197)–PE-Cy7 | 3D12 | BD Biosciences |
| CD27–PE | O323 | eBioscience |
| IgD–FITC | IA6-2 | eBioscience |
| CD24–PE-Cy7 | eBioSN3 (SN3 A5-2H10) | eBioscience |
| FoxP3–FITC | 236A/E7 | BD Biosciences |
