# Supplementary material for: Low‐grade chronic inflammation and immune alterations in childhood and adolescent cancer survivors: A contribution to accelerated aging?
Source: Cancer Med. 2021 Feb 19;10(5):1772–82. doi: 10.1002/cam4.3788 (PMC7940211; doi:10.1002/cam4.3788)
Supplement: Supplementary file 6 — Table S4 [file CAM4-10-1772-s007.docx]

**Table S4.** Comparison of antigen expression (MFI and percentages) on T cell subsets in cancer survivors and controls

|  |  |  | **T cells** | **CD4+ T cells** | **CD8+ T cells** |
| --- | --- | --- | --- | --- | --- |
| CD38 | **CS** | %  MFI | 34 [28.6-42.8]  707 [601-847] | 48.7 [42.5-59]  562 [510-565] | 7.5 [5.0-11.6]  1754 [962-3490] |
|  | **Controls** | %  MFI | 36.6 [34.3-46.9]*  579 [532-634]*** | 53 [48-61]  562 [461-625] | 7.4 [6.1-10.4]  1006 [763-1441]** |
| CD69 | **CS** | %  MFI | 1.0 [0.7-1.5]  1640 [1435-2235] | 0.7 [0.6-1.0]  922 [839-1097] | 1.8 [1.2-3.1]  1309 [1139-1580] |
|  | **Controls** | %  MFI | 0.9 [0.7-1.2]  1520 [1366-1649] | 0.7 [0.5-0.8]  897 [810-1020] | 1.8 [1.4-2.5]  1332 [1182-1461] |
| CD28 | **CS** | %  MFI | 87 [79.3-92.2]  3772 [3541-4201] | 99.3 [97.3-99.9]  3433 [3114-3706] | 74.3 [63.7-84.9]  3892 [3634-4328] |
|  | **Controls** | %  MFI | 88.5 [83.1-91.3]  3618 [3289-3828]* | 99.6 [97.9-99.8]  2959 [2749-3139]** | 76.4 [66.9-82.5]  3524 [3294-3683]** |
| CD57 | **CS** | %  MFI | 16.5 [11-23.2]  20850 [12443-28670] | 5.8 [4-7.5]  9202 [4231-18462] | 28.8 [18.4-38.8]  24227 [16627-33783] |
|  | **Controls** | %  MFI | 14.7 [10.1-20.7]  27360 [18886-34228]* | 5.0 [3.3-6.1]  8684[4651-25163] | 25.5 [20.0-37.6]  33687 [24152-41499]** |

Data are presented as medians [interquartile range] of percentages and MFI; *p<0.05; **p<0.01; ***p<0.001; CS, cancer surivors; MFI, mean fluorescence intensity.
