# Supplementary material for: Low‐grade chronic inflammation and immune alterations in childhood and adolescent cancer survivors: A contribution to accelerated aging?
Source: Cancer Med. 2021 Feb 19;10(5):1772–82. doi: 10.1002/cam4.3788 (PMC7940211; doi:10.1002/cam4.3788)
Supplement: Supplementary file 7 — Table S5 [file CAM4-10-1772-s005.docx]

**Table S5.** Relationship of selected lymphocyte subpopulations estimated from mRNA expression in whole blood with age in FHS and GTEx cohorts.

|  | **GTEx** | | **FHS** | |
| --- | --- | --- | --- | --- |
|  | **coefficient** | **p-value** | **coefficient** | **p-value** |
| B cell | -0.0002 | 0.004 | -0.0005 | <0.001 |
| T cell CD4+ memory | 0.00009 | 0.020 | -0.0008 | <0.001 |
| T cell CD4+ naive | -0.0001 | 0.100 | -0.0002 | <0.001 |
| T cell CD4+ (non-regulatory) | 0.00003 | 0.790 | -0.0005 | <0.001 |
| T cell CD4+ central memory | 0.0003 | 0.032 | -0.0001 | <0.001 |
| T cell CD4+ effector memory | 0.000006 | 0.069 | -0.00003 | <0.001 |
| T cell CD8+ naive | 0.00001 | 0.017 | -0.00001 | <0.001 |
| T cell CD8+ | 0.001 | 0.001 | -0.0006 | <0.001 |
| T cell CD8+ central memory | 0.001 | <0.001 | -0.0008 | <0.001 |
| T cell CD8+ effector memory | 0.001 | <0.001 | 0.00002 | <0.001 |
| Class-switched memory B cell | -0.0001 | 0.262 | 0.0000002 | 0.9 |

Data are shown as coefficients in the linear model (adjusted for sex), and p-values. GTEx, Genotypes and Phenotypes Project; FHS, Framingham Heart Study.
